# Interaction of Motor Training and Intermittent Theta Burst Stimulation in Modulating Motor Cortical Plasticity: Influence of BDNF Val66Met Polymorphism

**DOI:** 10.1371/journal.pone.0057690

**Published:** 2013-02-25

**Authors:** Mina Lee, Song E. Kim, Won Sup Kim, Jungyeun Lee, Hye Kyung Yoo, Kee-Duk Park, Kyoung-Gyu Choi, Seon-Yong Jeong, Byung Gon Kim, Hyang Woon Lee

**Affiliations:** 1 Department of Neurology, Ewha Womans University School of Medicine, Seoul, Korea; 2 Ewha Medical Research Institute, Seoul, Korea; 3 Department of Medical Genetics, Ajou University School of Medicine, Suwon, Korea; 4 Department of Neurology, Ajou University School of Medicine, Suwon, Korea; University of Toronto, Canada

## Abstract

Cortical physiology in human motor cortex is influenced by behavioral motor training (MT) as well as repetitive transcranial magnetic stimulation protocol such as intermittent theta burst stimulation (iTBS). This study aimed to test whether MT and iTBS can interact with each other to produce additive changes in motor cortical physiology. We hypothesized that potential interaction between MT and iTBS would be dependent on BDNF Val66Met polymorphism, which is known to affect neuroplasticity in the human motor cortex. Eighty two healthy volunteers were genotyped for BDNF polymorphism. Thirty subjects were assigned for MT alone, 23 for iTBS alone, and 29 for MT + iTBS paradigms. TMS indices for cortical excitability and motor map areas were measured prior to and after each paradigm. MT alone significantly increased the motor cortical excitability and expanded the motor map areas. The iTBS alone paradigm also enhanced excitability and increased the motor map areas to a slightly greater extent than MT alone. A combination of MT and iTBS resulted in the largest increases in the cortical excitability, and the representational motor map expansion of MT + iTBS was significantly greater than MT or iTBS alone only in Val/Val genotype. As a result, the additive interaction between MT and iTBS was highly dependent on BDNF Val66Met polymorphism. Our results may have clinical relevance in designing rehabilitative strategies that combine therapeutic cortical stimulation and physical exercise for patients with motor disabilities.

## Introduction

The neural circuitry in the human motor cortex commands highly sophisticated motor behaviors during goal-directed movements. It has been demonstrated that the motor map representation in the motor cortex is susceptible to changes in motor activity or experience [1], [2]. After injuries to the motor cortex, rehabilitative training reshapes the motor map reorganization, which is thought to play a role in motor functional recovery [3]. Therefore, motor training has been the mainstay of rehabilitative strategies to improve motor function after injuries such as stroke [4].

Transcranial magnetic stimulation (TMS) has been applied to the human motor cortex not only to measure motor cortical physiology but also to induce plastic changes in cortical neural circuit, and therapeutic potential of non-invasive cortical stimulation using repetitive TMS (rTMS) has been actively explored [5]–[7]. What would be more appealing is a hypothesis that the cortical stimulation combined with rehabilitative physical training may enhance efficacy of rehabilitation [8], [9]. Intermittent theta burst stimulation (iTBS), which are given as trains of low-intensity bursts at theta frequency, effectively facilitates motor cortical excitability long after the cessation of the stimuli [10], [11]. Therefore, the iTBS paradigm is regarded as a human equivalent of long-term potentiation of synaptic connectivity [12], [13]. The exact mechanism of iTBS-induced potentiation of the human cortical excitability and plasticity, however, remains to be fully understood. In regard to the therapeutic potential of cortical stimulation combined with rehabilitative training, it would be particularly intriguing to know whether iTBS can interact with motor training (MT) to produce additive effects on modulation of the motor cortical plasticity.

Brain derived neurotrophic factor (BDNF) is known to translate changes in neural activity into structural remodeling [14]. Secreted BDNF in response to neural activity modulates acute neural transmission [15] as well as induces long-term enhancement of synaptic transmission [16]. A single nucleotide polymorphism at nucleotide 196 of BDNF gene (Val to Met substitution at codon 66) affects activity-dependent secretion of BDNF [17]. It has been demonstrated that both experience-driven and iTBS induced human cortical plasticity is modulated by the BDNF polymorphism [11], [18].

The present study evaluated whether MT and iTBS paradigms could change the cortical excitability and representational motor map plasticity in healthy subjects. We were particularly interested in examining a potential interaction between the two paradigms in eliciting neuroplasticity in the motor cortical circuitry. We hypothesized that the potential interaction between MT and iTBS in the modulation of cortical physiology will also be dependent on BDNF Val66Met genotypes.

## Materials and Methods

### Subjects

Eighty-two healthy volunteers (mean age, 32.9 years old, 42 males and 40 females) were enrolled for this study. Thirty subjects were randomly assigned for MT alone, 23 for iTBS alone, and 29 for MT + iTBS combination paradigm. Entry criteria were age 20–40 years, right-handed, and without any neurological or psychiatric disorders. Right-handedness was determined on the basis of the Edinburgh questionnaire. The purposes and possible consequences of the study were fully provided, and written informed consents were obtained from all participating subjects, according to the protocol approved by the Ethical Committee of the Ewha Womans University Medical Center and ethical principles of the Declaration of Helsinki.

### Measurement of TMS indices

All subjects were comfortably seated in an armed chair and instructed to get as relaxed as possible. A blue surgery cap with a marked grid was tightly fitted around a subject's head and the central point was matched with the vertex position (defined as Cz by the 10–20 international system for EEG electrode). Single and paired pulse TMS were delivered through a focal figure of eight magnetic coil (10 cm diameter for each coil and 16.9 cm for the external head transducer) connected to a magnetic stimulator (MagPro Rapid Rate Magnetic Stimulator, Medtronic, Inc., Shoreview, MN, USA). Motor evoked potentials (MEPs) were recorded using surface EMG Ag-AgCl electrodes placed over the first dorsal interosseous (FDI) muscle in a belly-tendon montage. EMG raw signals were amplified, bandpass-filtered (5 Hz to 5 kHz), and recorded on a personal computer using data collection and averaging software (Toennies Neuroscreen Plus, Hoechberg, Germany). The intersection of the two wings of the coil was placed tangentially to the scalp with the handle pointing backward and laterally at a 30° to the mid-sagittal line, in order to induce the current from posterior to anterior and to optimally activate the corticospinal system trans-synaptically [19]. The TMS coil was placed flat on the skull over the optimal scalp positions to activate contralateral FDI muscles. We determined the optimal scalp position by moving the coil in 1 cm steps around the left primary hand motor area. A hot spot of the primary motor cortex (defined as M1) was determined as the cortical point with the lowest resting motor threshold (RMT), and was marked on the scalp with a soft pen. The RMT was defined as the minimal stimulus intensity required to induce at least five MEPs larger than 50 µV (peak to peak amplitude) out of 10 consecutive stimulations [20]. The recruitment curve was determined as MEP amplitudes at different intensities of 100%, 120%, and 140% RMT in each subject. Peak to peak amplitudes were measured during each trial, and a total of ten consecutive MEPs were later averaged at each stimulation intensity. In our study, the RMT was ranged from 57 to 73% of maximal output, higher than a previous report (Wassermann, 2002). The difference is possibly due to using different coil types with different magnetic power output, distance from coil to the cortical surface, skull thickness, cortical sulcal pattern, brain volume, ethnicity, etc [21]–[24]. Since 3 subjects (2 in MT and 1 in MT + ITBS experiments) had baseline RMTs higher than 72%, 140% RMT stimulation intensity was out of the maximum output (100%) capacity of the stimulator in these subjects. Therefore, the stimulation intensity was slightly compromised to the level with the 100% magnetic output (137∼139% of RMT). The maximal peripheral M response (Mmax) was measured by a stimulation of the ulnar nerve at the wrist at each session before the measurement of TMS indices, and absolute MEP amplitudes were expressed relative to the Mmax of the same session, as normalized MEP (nMEP). There was no significant difference between the Mmax values before and after any paradigm.

Paired-pulse stimulations for ICI and ICF were measured using well described paired pulse protocols at 2 ms for ICI and 15 ms for ICF [25]. The intensity of the conditioning stimulus was 70% RMT, and the intensity of the test stimulus was adjusted to produce MEPs of approximately 1 mV peak-to-peak amplitude in the resting FDI. The inter-trial interval was set as 5 seconds. The amplitude of the conditioned MEP was expressed relative to the unconditioned MEP for each ISI.

### Cortical motor mapping

To determine the optimal scalp position for eliciting MEPs from the target muscle, which was the FDI in our study, each stimulation site was guided by a coordinate system (1×1 cm width) on the cap with a marked grid. After a hot spot was determined where the RMT was the lowest, we mapped the brain surface area showing a positive response (at least 3 MEPs ≥ 50 µV out of 10 trials) at 110% RMT intensity on each stimulation site across the surface coordinate. The procedure was continued until each stimulation site with positive response was surrounded by negative sites. The number and locations of positive sites were recorded as the FDI representational cortical motor map areas in each subject [11], [18]. Topographic map based on the percentage data of subjects with positive response at each location was constructed by an interpolated color surface map using the MATLAB 7.3 (MathWorks, Natick, MA, USA). For color coding, deep red color areas represented motor cortical regions with MEP responses elicited in 100% subjects, and those with no MEP responses in any subject were colored in deep blue.

### Motor performance test

For motor performance test, subjects were seated comfortably on a chair, with right hand placed on a desk. Subjects were asked to perform with their right hand motor tasks that involved the FDI muscle. The left hand remained on the table and was kept relaxed. For finger tapping, flexion and extension of the right index finger were repeated. Subjects were instructed to tap a linear strain gauge (model MLP-25, Transducer Techniques, PAR, Lutz, Florida, USA) as fast as possible with their index finger. The number of taps was counted over a 15-second interval. Thirty trials lasting 15 minutes were performed, with at least 15-second rest in between each trial. The total number of finger tapping counts taken to complete the test was recorded, and the mean number per minute was used for data analysis.

### MT alone, iTBS alone, and combined MT plus iTBS paradigms

For the MT paradigm, the subjects received MT immediately after the baseline measurement of TMS indices and motor performance. For MT paradigm, each subject was asked to press the 1 and 3 keys alternatively on a keyboard with the right index finger as fast as possible for 15 seconds, followed by a 15-second break, which was repeated ten times, followed by a 3-min break. After the MT paradigm, sham stimulation (for MT alone experiment) was applied with the magnetic coil rotated through 90° at the hot spot using the same stimulus parameters as for the active iTBS [26]. For the iTBS paradigm, we applied iTBS on a hot spot applying 50 Hz three-pulse bursts repeated at 200 ms intervals (5 Hz) in short trains with 80% subthreshold of RMT [10]. A train of TBS lasting 2 seconds was repeated every 10-second for a total of 190 seconds (600 pulses). For stimulation of M1, the coil was placed over the hot spot with the handle directed posterolaterally. After the MT or iTBS paradigm, we measured TMS indices and the FDI representational cortical map areas using the RMT values measured at the same session. After all TMS measurements were done, motor performance was tested again. For the combined MT + iTBS paradigm, the subjects were asked to proceed the MT session after initial baseline TMS measurement followed by iTBS on the hot spot.

### BDNF Val66Met genotyping

Genomic DNA was extracted from peripheral blood samples using the QIAamp DNA Blood Mini Kit (Qiagen). Exon 2 region of the *BDNF* gene was amplified using the polymerase chain reaction (PCR) with the following primer set: 5′-CCTGCAGAATGGCCTGGAATTAC-3′ and 5′-TGCCGTTACCCACTCACTAATACTG-3′. The PCR reaction contained 200 nM dNTPs, 200 nM of each primer, 1× PCR buffer, and 2.5 U of LA Taq polymerase (Takara). The conditions for amplification were as follows: initial denaturation at 95 °C for 1 min; followed by 20 s at 95 °C, 30 s at 60 °C, and 30 s at 72 °C for 35 cycles; with a final extension at 72 °C for 2 min. The amplified PCR products (573 bp) were separated in a 1.5% agarose gel stained with ethidium bromide, and then sequenced using an automatic sequencer, the ABI 3730xl DNA analyzer (Applied Biosystems) in both directions with the same primers used for PCR amplification. Sequences were analyzed by comparison with the corresponding wild-type reference GBA sequence (GenBank: NG_011794.1). A single nucleotide polymorphism (SNP) at nucleotide 196 (G to A), which results in the substitution of an amino acid at codon 66 (Val to Met), was determined in each allele, and all the participating subjects were categorized into Val/Val (wild type), Val/Met (heterozygote), and Met/Met (homozygote) genotypes.

### Data analysis

All statistical analyses were performed by SPSS 12.0 statistical software (Chicago, IL, USA). All numerical data were presented as mean ± standard error of mean (SEM). Statistical analyses were carried out with the assumption of 0.05 as the level of significance. An unpaired t-test or one way ANOVA was used to compare differences in the demographic variables between the subjects in different experimental paradigms or in different genotype groups, respectively. A one way ANOVA was also used to compare the differences in the baseline TMS indices, behavioral performances, and motor map areas between different genotype groups. A statistical significance of changes in measured variables before and after the experimental paradigms (MT alone, iTBS alone, or MT + iTBS) was tested using a paired t-test. The effects of genotypes on changes in the measured variables before and after the experimental paradigms were tested using a repeated measure two-way ANOVA. A general linear model (GLM) was used to test the statistical significance of differences in the measured variables between MT, iTBS, and MT + iTBS paradigms (paradigm effects). The effects of between-subjects factors (genotype effects) were also examined and possible interactions between the two (paradigm Х genotype) were tested using the GLM.

## Results

### Prevalence of BDNF Val66Met polymorphism in study participants

BDNF genotyping showed that 31.7% of all the study participants (26/82) have a wild type Val/Val allele, 47.6% (39/82) have one Val allele substituted by Met (Val/Met), and 20.7% (17/82) have both Val alleles substituted by Met (Met/Met). Compared to the allelic distributions in the Caucasian population [27], Korean subjects showed higher prevalence of Met alleles, which is similar to that of other Asian ethnic groups [28], [29]. The allelic distribution was not significantly different between the three different experimental groups (MT alone, iTBS alone and MT + iTBS) (Table 1). Mean ages and sex ratios were comparable between the different genotype groups, as well as between the subject groups participating in different experiments (Table 1).

**Table 1:** Demographic variables and frequency of BDNF Val66Met polymorphism in the study participants.

Experiment	MT				iTBS				MT+iTBS			
Genotype	Val/Val	Val/Met	Met/Met	Subtotal	Val/Val	Val/Met	Met/Met	Subtotal	Val/Val	Val/Met	Met/Met	Subtotal
Age	33.6±9.4	30.3±9.4	35.3±8.0	33.1±9.2	29.6±3.6	32.5±4.7	31.3±5.3	31.9±4.4	35.2±11.2	34.0±10.0	29.0±8.2	33.7±10.0
M∶F	6∶5	5∶5	5∶4	16:14	4∶2	4∶9	2∶2	10∶13	4∶5	10∶6	2∶2	16∶13
Number (%)	11 (36.7%)	10 (33.3%)	9 (30.0%)	30 (100%)	6 (26%)	13 (56.6%)	4 (17.6%)	23 (100%)	9 (31.0%)	16 (55.2%)	4 (13.8%)	29 (100%)

MT: motor training, iTBS: intermittent theta burst stimulation, M: male, F: female.

### MT-induced changes in motor cortical physiology

**Figure 1:**
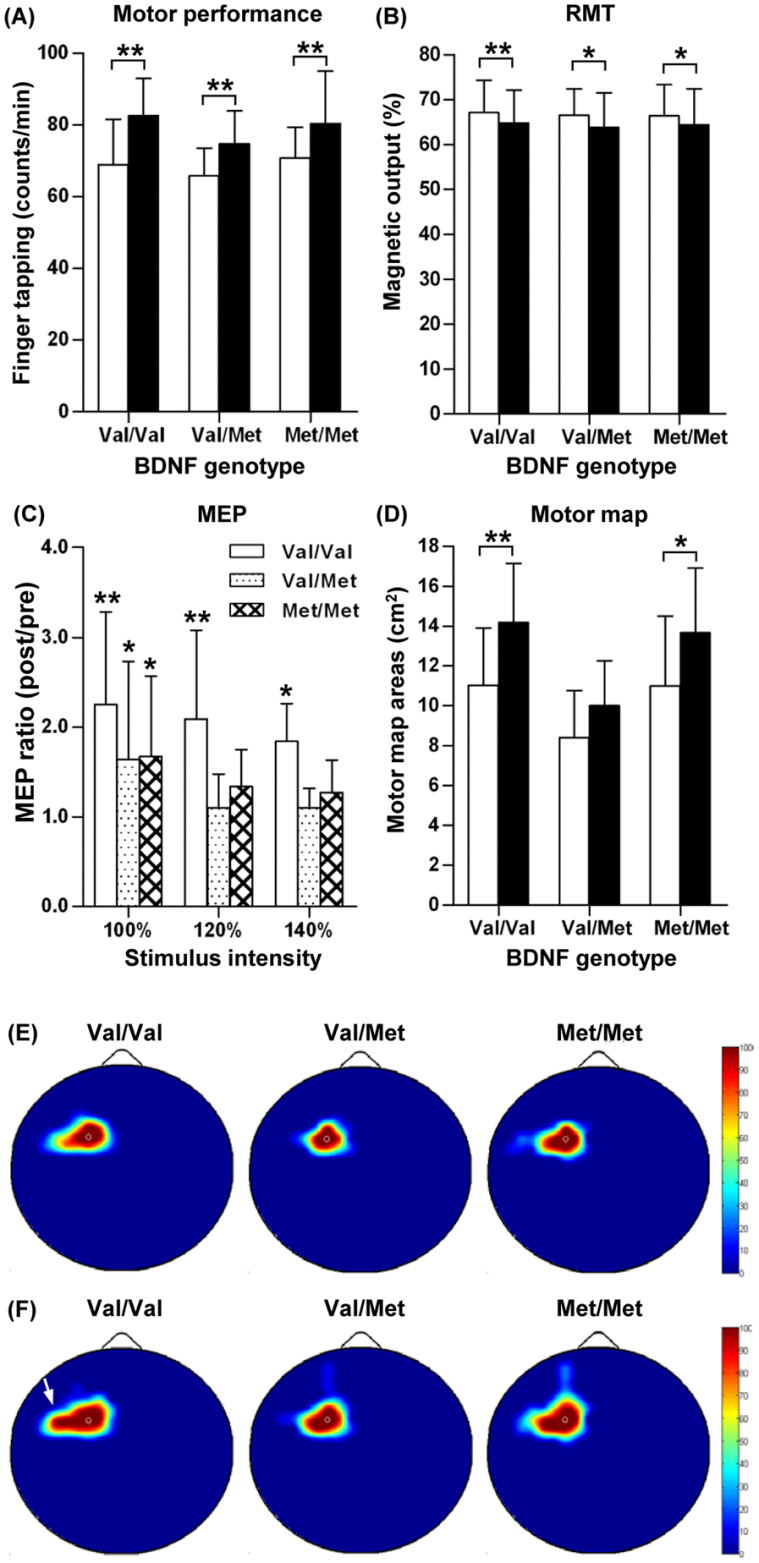
Changes in motor cortical physiology by motor training alone. Motor behavior performances and motor cortical physiology variables were measured before and after motor training (MT). Subjects were grouped by BDNF Val66Met genotypes. (**A**) Motor behavior performances measured by the numbers of finger tapping in one minute. (**B**) Resting motor threshold (RMT) measured as a percentage of magnetic output. (**C**) Motor evoked potentials (MEP) elicited by different stimulation intensities. MEP changes are presented as a ratio of post- versus pre-MET values for the sake of clarity. (**D**) Motor map areas. White bars represent values before MT and black bars values after MT in (**A**), (**B**), and (**D**). * and ** denote *p*<0.05 and *p*<0.01 by a paired t test, respectively. (**E, F**) Color-coded MEP response frequency maps were generated before (**E**) and after (**F**) MT by plotting and compiling motor cortical regions eliciting MEP responses at 110% RMT intensity from the subjects in the same genotype groups. Deep red color areas represent motor cortical regions eliciting MEP responses in 100% subjects and those with no MEP responses in any subject are colored in deep blue. A white arrow in the post-MT map of Val/Val genotype group points to the region of prominent lateral map expansion.

Baseline TMS indices, such as RMT, MEP amplitudes at different stimulation intensities, ICI, and ICF, were not significantly different between the three genotype groups (Table 2). Motor performance measured before MT was also not significantly different among the three genotype groups. The MT session significantly improved the motor performances (measured by the number of finger tapping per minute) in all genotype groups by a pair-wise comparison (Fig. 1A). The same indices of motor cortical excitability were measured again after the MT session (Table 2). The mean RMT values in all genotype groups significantly decreased as compared to that of the baseline (Fig. 1B), indicating that the MT session effectively increased the motor cortical excitability irrespective of genotype. MEP amplitudes at 100% RMT stimulus intensity were significantly increased after MT in all genotype groups (Fig. 1C). The extent of MEP increase (post/pre ratio) was greater in Val/Val than Val/Met or Met/Met groups, but the genotype effect was not statistically significant. MT-induced increases in MEP at 120% and 140% RMT were statistically significant only in Val/Val genotype group (Fig. 1C). However, there was no significant influence of BDNF Val66Met genotype on MT-induced changes in MEP amplitudes at these stimulus intensities. ICI or ICF values were not significantly changed by the MT alone paradigm (Table 2). We also measured motor map areas before and after the MT session. The baseline motor map areas were not significantly different between the different genotype groups. The mean total motor map areas were increased after MT session compared to the baseline in all the genotype groups (Table 2). The extent of increase in total motor map areas was statistically significant in Val/Val and Met/Met groups by a pair-wise comparison, and there was a significant genotype influence (*F_(2,27)_* = 5.398, *P* = 0.011). In order to evaluate the qualitative differences before and after the MT session, the motor cortical regions eliciting MEP responses were plotted in each subject and compiled across the subjects in the same genotype group to generate a map of MEP response frequency (Fig. 1E, F). Intriguingly, cortical regions eliciting MEP responses expanded mainly in the lateral direction relative to M1 in the subjects with the Val/Val genotype (Fig. 1F). The subjects with Val/Met or Met/Met genotypes also showed motor map expansion, but without apparent directional specificity.

**Table 2:** Motor performance and measured TMS indices before and after motor training (MT), intermittent theta burst stimulation (iTBS), or MT + iTBS.

Experiment		MT only			iTBS only			MT + iTBS		
Genotypes		Val/Val	Val/Met	Met/Met	Val/Val	Val/Met	Met/Met	Val/Val	Val/Met	Met/Met
Finger tapping (no./min)	Pre	68.91±12.69	65.08±7.73	70.87±8.45	62.72±11.51	63.50±8.43	56.79±5.31	65.43±9.50	67.50±11.77	58.05±9.50
	Post	82.48±10.56	74.62±9.23	80.39±14.53	71.54±14.18	70.10±6.85	67.50±5.30	79.03±5.86	78.44±12.98	74.46±7.67
	Ratio	1.21±0.20	1.15±0.05	1.12±0.08	1.14±0.13	1.10±0.07	1.12±0.12	1.23±0.19	1.17±0.12	1.29±0.14
RMT (%)	Pre	67.09±6.12	66.50±5.28	66.33±7.00	65.00±3.84	64.61±5.93	66.75±4.57	67.66±5.63	63.75±7.91	66.50±2.88
	Post	64.72±7.39	63.80±7.67	64.33±8.06	60.00±3.28	60.38±5.47	62.75±4.27	59.66±6.18	58.18±8.36	62.50±4.79
	Ratio	0.96±0.03	0.95±0.04	0.96±0.02	0.92±0.01	0.93±0.03	0.94±0.01	0.88±0.03	0.91±0.04	0.93±0.04
nMEP at 100% RMT	Pre	3.82±1.41	4.38±1.29	3.43±1.48	4.72±1.04	4.68±1.24	6.18±2.02	3.84±1.02	4.83±1.41	4.03±1.24
	Post	8.58±2.58	7.29±2.99	5.58±1.71	8.54±2.01	7.58±1.61	7.21±1.53	9.72±1.44	7.80±2.42	5.31±1.27
	Ratio	2.25±1.03	1.64±1.09	1.67±0.85	1.83±0.75	1.62±0.52	1.18±0.25	2.57±0.42	1.82±0.36	1.37±0.71
nMEP at 120% RMT	Pre	65.32±29.14	77.09±54.11	44.63±37.68	75.08±7.91	66.14±26.58	82.13±29.78	59.42±14.81	68.03±20.03	66.24±27.01
	Post	135.04±42.42	88.12±56.61	68.12±39.72	129.27±14.33	85.04±27.49	100.23±22.04	163.13±43.32	104.98±30.78	92.37±28.21
	Ratio	2.09±0.99	1.11±0.28	1.34±0.37	1.91±0.34	1.29±0.23	1.22±0.23	2.63±0.46	1.56±0.63	1.43±0.38
nMEP at 140% RMT	Pre	125.62±84.11	129.03±83.98.	107.49±53.18	115.39±9.10	118.84±30.21	107.01±27.02	120.79±71.48	138.02±46.67	120.27±38.06
	Post	231.04±85.42	153.12±67.47	127.58±56.76	208.23±38.59	143.82±38.18	136.17±17.53	271.31±93.16	175.22±45.41	158.08±43.07
	Ratio	1.38±0.49	1.02±0.25	1.12±0.24	1.81±0.20	1.29±0.21	1.14±0.02	2.13±0.59	1.38±0.36	1.28±0.38
ICI (ratio)	Pre	0.44±0.23	0.47±0.34	0.64±0.30	0.43±0.18	0.37±0.14	0.40±0.10	0.36±0.09	0.36±0.14	0.36±0.18
	Post	0.38±0.19	0.35±0.19	0.49±0.17	0.44±0.21	0.41±0.14	0.35±0.12	0.33±0.13	0.34±0.16	0.37±0.13
	Ratio	1.06±0.59	0.91±0.59	0.90±0.41	1.05±0.38	1.21±0.45	0.97±0.58	0.98±0.44	1.00±0.49	1.17±0.54
ICF (ratio)	Pre	2.01±1.03	2.27±1.31	2.35±3.28	2.50±0.71	2.54±0.86	2.24±0.76	2.22±1.83	2.49±1.06	2.51±0.69
	Post	2.39±2.14	2.35±1.51	2.36±1.53	2.56±0.71	3.13±1.66	2.46±0.59	2.69±1.39	2.57±1.95	2.26±0.44
	Ratio	1.28±1.14	1.08±0.31	1.06±0.70	1.04±0.21	1.22±0.40	1.12±0.13	1.54±1.00	1.05±0.37	0.92±0.15
Motor map areas (cm^2^)	Pre	11.02±2.89	8.40±2.36	11.03±3.50	10.0±3.63	10.84±2.40	9.50±1.73	10.11±1.36	8.68±2.21	10.51±3.00
	Post	14.18±2.96	10.00±2.26	13.66±3.24	14.33±6.18	14.15±4.66	14.5±4.79	20.44±1.23	10.37±2.02	17.25±3.20
	Ratio	1.31±0.20	1.25±0.37	1.28±0.23	1.48±0.10	1.45±0.24	1.30±0.17	2.05±0.31	1.25±0.36	1.71±0.43

MT: motor training, iTBS: intermittent theta burst stimulation, no.: number, min: minute, RMT: resting motor threshold, nMEP: normalized motor evoked potential, ICI: intracortical inhibition, ICF: intracortical facilitation, Pre: before MT or MT + iTBS, Post: after MT or MT + iTBS, Ratio: ratio of Post divided by Pre values.

### iTBS-induced changes on motor cortical physiology

**Figure 2:**
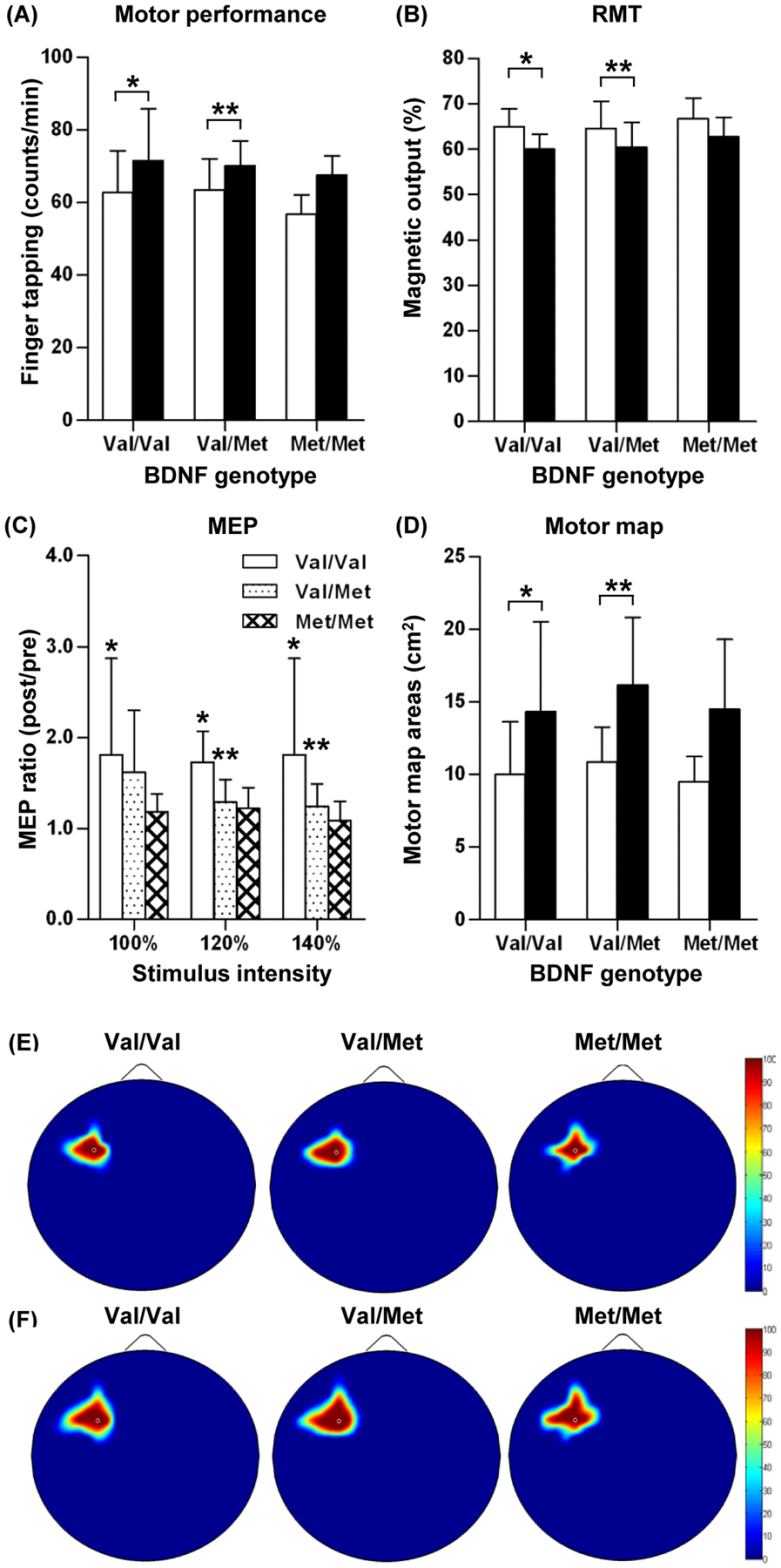
Changes in motor cortical physiology by intermittent theta burst stimulation alone. Motor behavior performances and motor cortical physiology variables were measured before and after the intermittent theta burst stimulation (iTBS) paradigm. Subjects were grouped by BDNF Val66Met genotypes. (**A**) Motor behavior performances measured by the numbers of finger tapping in one minute. (**B**) Resting motor threshold (RMT) measured as a percentage of magnetic output. (**C**) Motor evoked potentials (MEP) elicited by different stimulation intensities. MEP changes are presented as a ratio of post- versus pre-iTBS values for the sake of clarity. (**D**) Motor map areas. White bars represent values before iTBS and black bars values after iTBS in (**A**), (**B**), and (**D**). *, **, and *** denote *p*<0.05, *p*<0.01, and *p*<0.001 by a paired t test, respectively. (**E, F**) The same color-coded MEP response frequency maps as in Fig. 1(**E, F**) were generated before (**E**) and after (**F**) iTBS.

In the next experiment, a group of subjects received iTBS to the primary motor cortex without MT. The iTBS paradigm also enhanced motor performance in all the genotype groups (Fig. 2A). The mean RMT values significantly decreased in Val/Val and Val/Met, but not in the Met/Met group after the iTBS paradigm (Fig. 2B). The genotype effect on the decrease in RMT by iTBS, however, was not statistically significant. The iTBS paradigm tended to increase MEP amplitudes at all the three stimulus intensities regardless of genotypes (Fig. 2C). At 100% RMT intensity, the increases in MEP amplitudes were statistically significant in Val/Val genotype group, but not in the other groups. At 120 and 140% intensities, MEP amplitudes increased significantly in Val/Val and Val/Met groups. The effects of genotype on the increases in MEP amplitudes, however, were not significant in any stimulus intensity by repeated measures two-way ANOVA. ICI or ICF values were not significantly changed by iTBS alone (Table 2). The motor map also significantly expanded after the iTBS paradigm in the Val/Val and Val/Met groups, but not in the Met/Met group without significant genotype influence (Fig. 2D) (Table 2). We also generated MEP frequency map on the motor cortex. Comparison between pre and post iTBS paradigm showed some degree of motor map enlargement in all the genotype groups (Fig. 2E, F). The motor map expansion did not seem to be limited to any direction. Therefore, the directional specificity was not obvious compared to the motor map changes in MT alone group (Fig. 1E, F).

### Changes in motor cortical physiology induced by combination of MT and iTBS

**Figure 3:**
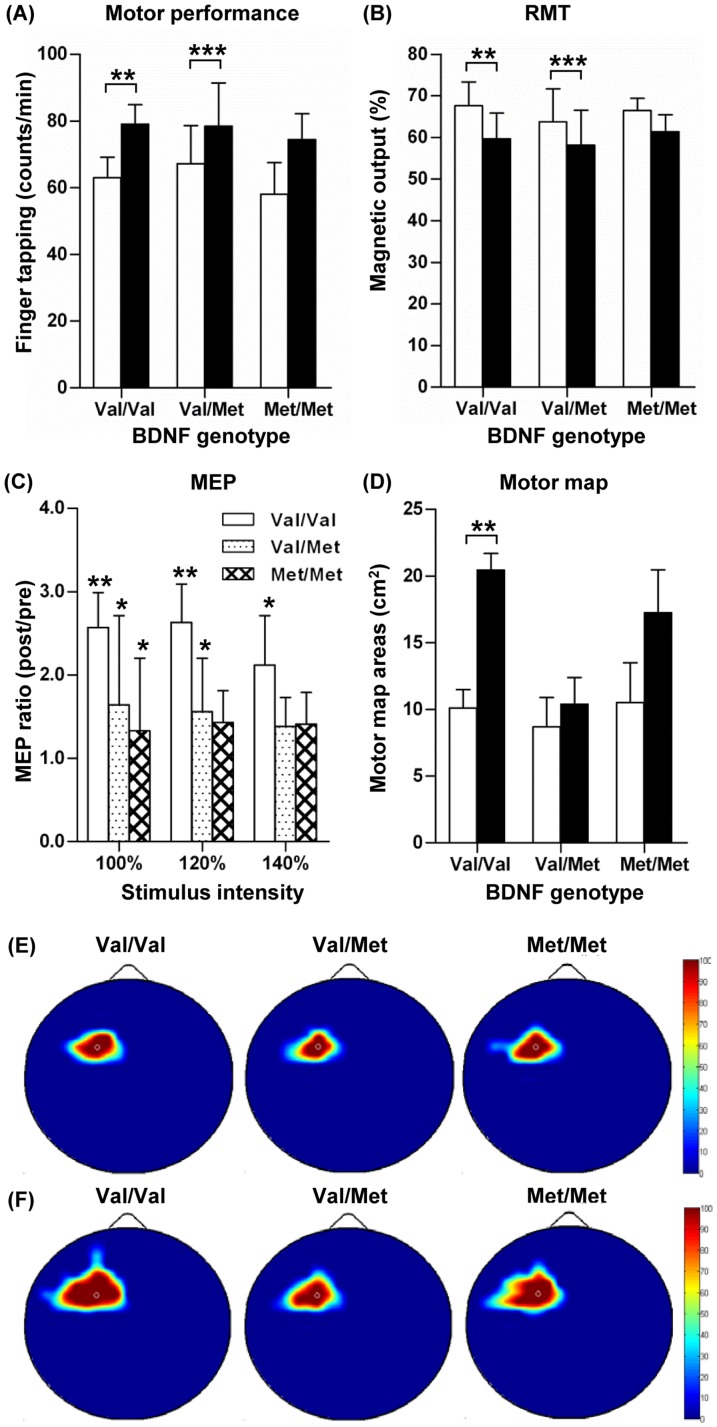
Changes in motor cortical physiology by motor training plus intermittent theta burst stimulation paradigm. Motor behavior performances and motor cortical physiology variables were measured before and after motor training (MT) immediately followed by intermittent theta burst stimulation (iTBS). Subjects were grouped by BDNF Val66Met genotypes. (**A**) Motor behavior performances measured by the numbers of finger tapping in one minute. (**B**) Resting motor threshold (RMT) measured as a percentage of magnetic output. (**C**) Motor evoked potentials (MEP) elicited by different stimulation intensities. MEP changes are presented as a ratio of post- versus pre-MT + iTBS values for the sake of clarity. (**D**) Motor map areas. White bars represent values before MT + iTBS and black bars values after MT + iTBS in (**A**), (**B**), and (**D**). *, **, and *** denote *p*<0.05, *p*<0.01, and *p*<0.001 by a paired t test, respectively. (**E, F**) The same color-coded MEP response frequency maps as in Fig. 1(**E, F**) were generated before (**E**) and after (**F**) iTBS.

The third group of subjects received the MT after a baseline measurement of TMS indices. Then, iTBS was applied to the primary motor cortex, which lasted 190 seconds immediately after MT session. The baseline motor performances, TMS indices, and the total map areas were not significantly different between the different genotype groups (Table 2). The MT + iTBS paradigm also enhanced motor performance in all the genotype groups with statistical significance in Val/Val and Val/Met groups (Fig. 3A). The mean RMT values significantly decreased in Val/Val and Val/Met, but not in the Met/Met groups after the MT + iTBS paradigm (Fig. 3B). However, there were no significant genotype effects either on the changes in motor performance or RMT values. MEP amplitudes increased at 100% RMT stimulus intensity in all the groups (Fig. 3C). At 120% RMT, MEP amplitudes were significantly increased in the Val/Val and the Val/Met groups. At 140% RMT, MT + iTBS led to a significant increase in MEP amplitudes only in the Val/Val group. However, there was no significant influence of BDNF Val66Met genotype on the changes in MEP amplitudes by MT + iTBS at all the stimulus intensities. ICI or ICF values were not significantly changed by MT + iTBS (Table 2). The motor map also expanded after MT + iTBS in all the genotype groups (Table 2). The greatest increase in the total motor map areas was observed in the Val/Val group (Fig. 3D), attaining almost a 100% expansion, when compared to that of the baseline. In contrast, differences in the total motor map before and after the MT + iTBS paradigm were not significantly different in the Val/Met or Met/Met groups, resulting in a significant genotype effect on the changes in the total motor map areas induced by MT + iTBS (*F_(2,26)_* = 40.510, *P*<0.001). In the compiled MEP frequency maps (Fig. 3E, F), the extent of motor map enlargement looked more obvious than that in MT alone or iTBS alone experiments especially in Val/Val subjects. Notably, the motor map expansion in the Val/Val group by the MT + iTBS paradigm occurred not only in the lateral but also to the anterior and medial directions in the MEP response frequency map, resembling the map expansion pattern in the iTBS paradigm (Fig. 2E, F).

### Quantitative comparison of changes in motor cortical physiology between the three different paradigms

**Figure 4:**
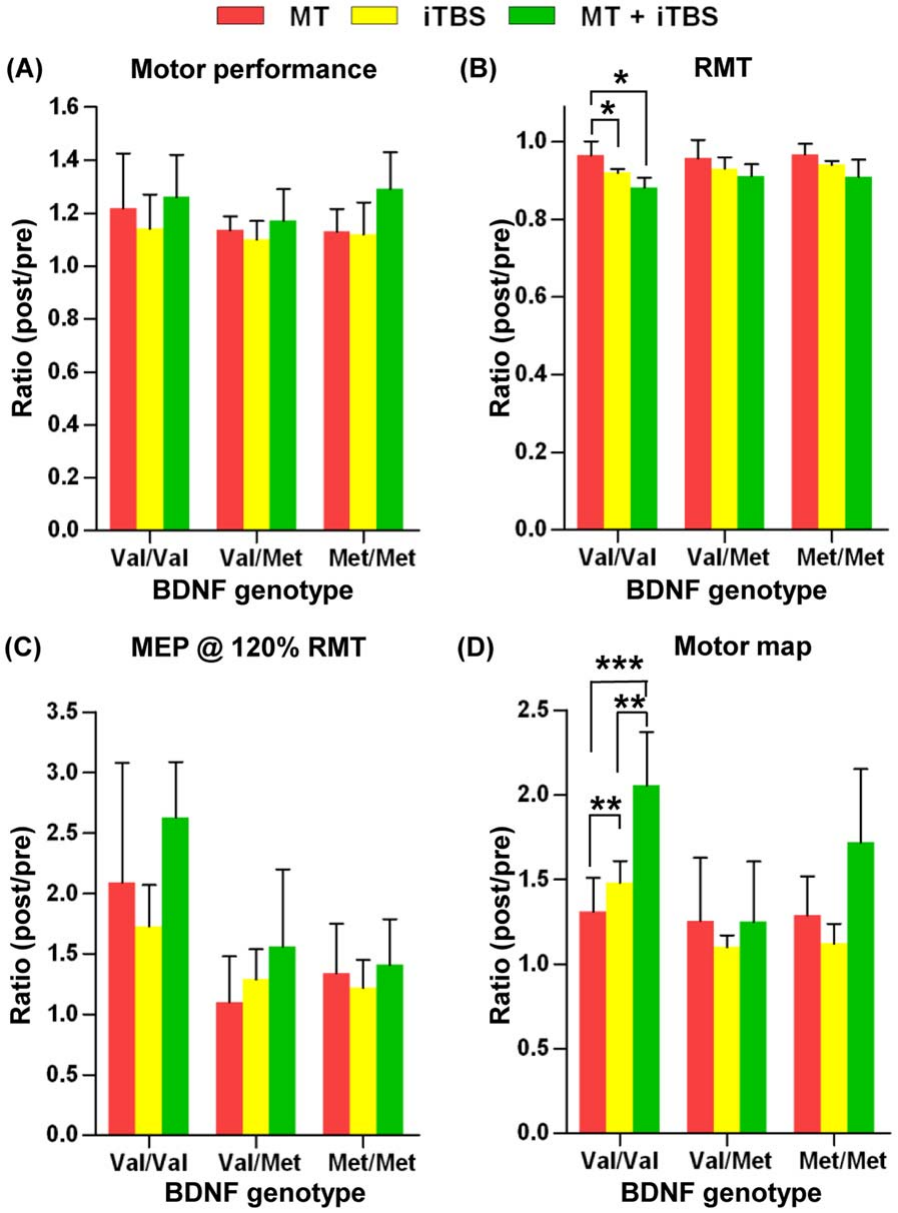
Quantitative comparison of changes in motor cortical physiology by three different paradigms. Red, yellow, and green bars represent ratios of post/pre-motor training (MT), post/pre-intermittent theta burst stimulation (iTBS), and post/pre MT + iTBS values in motor behavior performances and motor cortical physiology variables, respectively. Subjects were grouped by BDNF Val66Met genotypes. (**A**) Motor behavior performances measured by the numbers of finger tapping in one minute. (**B**) Resting motor threshold (RMT) measured as a percentage of magnetic output. (**C**) Motor map areas. *, **, and *** denote *p*<0.05, *p*<0.01, and *p*<0.001 by a general linear model two way ANOVA followed by a *posthoc* Bonferroni test.

To determine potential interactions between MT and iTBS in the modulation of motor cortical excitability and map plasticity, the extent of changes (post/pre ratio) in motor performance, cortical excitability indices, and motor map areas were directly compared between MT alone, iTBS alone and MT + iTBS paradigms using GLM statistics (Fig. 4). The MT + iTBS paradigm improved motor performance greater than MT or iTBS alone groups (paradigm factor; *F_(2,73)_* = 5.631, *P* = 0.006) (Fig. 4A). However, *posthoc* analysis in each genotype group did not reveal a significant difference, and accordingly, there was no significant genotype effect. The extent of RMT reduction was significantly influenced by different paradigms (paradigm factor; *F_(2,73)_* = 20.760, *P*<0.001) (Fig. 4B). *Posthoc* analysis revealed that the RMT reduction was significantly greater only in Val/Val genotype with iTBS alone or MT + iTBS paradigms than with MT alone, but there was no significant difference in the extent of RMT reduction between iTBS and MT + iTBS paradigms. However, BDNF genotypes did not significantly influence the extent of RMT reduction with any paradigm. The increases in MEP amplitude at all stimulation intensities were not significantly dependent on different paradigms. The genotype effect was significant only at intensity of 120% RMT (genotype factor; *F_(2,73)_* = 3.147, *P* = 0.049 at 120%) (Fig. 4C), indicating that subjects with Val/Val genotype increases MEPs at 120% RMT stimulus intensity in response to any of the three paradigms. The extent of motor map expansion was markedly influenced by different paradigms (paradigm factor; *F_(2,73)_* = 6.100, *P* = 0.004) (Fig. 4D). The iTBS paradigm expanded motor map areas significantly more than MT alone, and the motor map expansion was significantly greater by MT + iTBS than that either by MT or iTBS alone paradigms, indicating that MT and iTBS interact additively to expand motor cortical map plasticity. These differences between the different paradigms were significant only in the Val/Val group. Therefore, the genotype effect was highly significant (genotype factor; *F_(2,73)_* = 8.291, *P* = 0.001, genotype Х paradigm; *F_(4,73)_* = 4.117, *P* = 0.005), indicating that the interaction between MT and iTBS in modulating motor map plasticity is highly dependent on BDNF genotype.

## Discussion

Our experiments revealed the following major points; 1) both MT alone and iTBS alone paradigms significantly increased the motor cortical excitability and expanded the motor map areas, 2) when combined, MT and iTBS paradigms interacted additively in the modulation of the motor cortical excitability and the representational map plasticity, and 3) the interaction between MT and iTBS paradigms on motor map plasticity was highly dependent on BDNF Val66Met polymorphism.

It is known that MT increases the motor cortical excitability and enhances the cortical plasticity measured by motor map representation [30]–[33]. As previously reported in the previous studies [10], [34], we found that the iTBS paradigm also significantly modulated motor cortical physiology. The extent of increases in the MEP amplitude was quite comparable to that by MT alone. However, the amount of RMT reduction tended to be greater by iTBS than by MT alone, and the difference between the paradigms was significant in Val/Val genotype group (Fig. 4B). Expansion of motor map areas was slightly (1.31 ± 0.20 vs. 1.48 ± 0.10 fold), but significantly greater by iTBS than by MT alone only in Val/Val genotype group. These results indicate that the iTBS paradigm induces slightly (modestly at most) greater changes in motor cortical physiology than MT alone does, but this difference was evident only in Val/Val genotype group. A notable difference was the directional pattern of motor map expansion. The directional specificity of motor map expansion in the iTBS paradigm was not as prominent as in the MT experiment, where the motor map expanded preferentially to the lateral direction (Fig. 1E, F). It remains to be determined exactly how directionally specific motor map expansion occurred following MT. It is conceivable that spatial influence of the two paradigms may differ. Finger movements during MT session involve several muscles functioning for the flexion and extension of the index finger including FDI. Therefore, it is likely that the motor map of the FDI muscle may be preferentially expanded to the areas where the motor maps of other related muscles are represented. In contrast, possible effects of iTBS to a hot spot for the FDI muscle would spread to adjacent regions in a concentric manner without spatial preferences. Evaluating motor map expansion after MT involving a different muscle set would be helpful to corroborate the above notion.

In the current study, a combination of MT and iTBS resulted in enhanced motor cortical excitability compared to either MT or iTBS alone. The MT + iTBS paradigm improved finger tapping speed most, and the amount of RMT reduction was the greatest by combination of MT and iTBS. In addition, the increases in MEP amplitudes tended to be highest by MT + iTBS in Val/Val genotype. The additive effect of MT + iTBS was even more pronounced on the motor map expansion in Val/Val genotype group (Fig. 4D). The quantitative potentiation of iTBS induced plasticity by combination of preceding MT suggest a possibility that MT played a role in inducing ‘metaplasticity’ [35] in a way that changing activity in the motor cortex by MT affected susceptibility of motor cortical neurons to subsequent iTBS paradigm. The metaplasticity can be explained by alterations of synaptic (pre- and/or post-synaptic) efficacy [36] or calcium dynamics induced by prior activity [37]. It has been shown that outcomes of rTMS can be governed by the state of spontaneous neural activity [38]. Furthermore, priming activation of motor cortex may change the direction of aftereffects by subsequent magnetic stimulation in a homeostatic manner [39]–[41]. For example, preceding phasic finger movements reversed the aftereffects by iTBS to be inhibitory rather than facilitatory [42]. Our finding that MT potentiated the iTBS-induced cortical plasticity, therefore, may be more consistent with non-homeostatic metaplasticity [43]–[45] rather than following the rule of homeostatic mechanisms. According to the calcium dynamics theory [37], the MT paradigm in our study may not affect the amount of calcium entry induced by subsequent iTBS paradigm, yet may be sufficient to activate the intracellular calcium signaling cascade, rendering neurons in the motor cortex able to generate potentiated responses to a next wave of calcium entry upon subsequent iTBS paradigm. In this regard, differences in the detailed nature of motor tasks between the MT paradigm in our study and the similar finger movements employed in the previous study [42] may have led to differences in calcium entry upon subsequent stimulation. Our MT paradigm consists of 10 rounds of 15-second finger movements and 15-second rest, whereas in the study of Iezzi et al. (2008), the duration of rest between movements was only 5 second. The shorter rest duration may not allow the amount of available calcium to fully recover, and the altered calcium entry upon subsequent iTBS may change the direction of aftereffects of iTBS.

Changes in motor cortical physiology by different paradigms were influenced by BDNF Val66Met polymorphism. The extent of RMT reduction was significantly greater by iTBS or MT + iTBS compared to MT only in Val/Val group (Fig. 4B). The mean increases in MEPs by any paradigm tended to be greater in Val/Val than the other two genotype groups, and there was a significant genotype effect on MEP increases at 120% RMT stimulation intensity. The most obvious genotype effect was observed on the interaction between MT and iTBS in the enhancement of motor map plasticity where motor map expansion was markedly greater in the combined paradigm than MT or iTBS alone only in Val/Val group (Fig. 4D). It has been shown that substitution of valine with methionine at codon 66 of BDNF gene affects depolarization-induced synaptic secretion of BDNF [17], [46]. BDNF released in response to synaptic activity enhances both a rapid and a long-term synaptic transmission [15], [16]. Therefore, defective BDNF secretion by Val/Met substitution can lead to remarkable effects on motor cortical physiology. BDNF is also involved in gating the sodium channel and inducing action potentials [47], suggesting that motor cortical excitability may also be influenced by the BDNF polymorphism. Furthermore, BDNF signaling can leave molecular signatures to activated synapses by phosphorylating glutamate receptors [48], [49], which in turn significantly modulates receptor function. It could be conceivable that the MT paradigm stimulates BDNF release, the released BDNF enhances NMDA receptor function by phosphorylating NMDA receptor subunits, and the NMDA receptors with increased activity produce the additive interaction between MT and subsequent iTBS paradigms. In this scenario, defective BDNF genotype would interfere with the preceding MT-induced release of BDNF, and insufficient NMDA receptor activation by reduced BDNF signaling would lead to a failure of significant potentiation of motor map plasticity by iTBS paradigm. Thus, BDNF can play an important modulatory role in metaplasticity by regulating postsynaptic mechanisms [36]. Together, our data suggest that BDNF polymorphism influences MT or iTBS induced changes in motor cortical physiology, and intact BDNF function may be required for the additive interaction between the MT and iTBS paradigms in the enhancement of motor map plasticity.

The conclusion that MT and iTBS interact with each other in the modulation of motor cortical physiology bears an important clinical implication in the recovery of function after CNS injury. Exercise or motor training has long been provided to patients with neurological motor disability as a sole therapeutic measure to improve motor function. Novel rehabilitative approaches are awaited to be developed to achieve more significant functional recovery [50], [51]. Therapeutic cortical stimulation with rTMS has been applied to modulate the cortical plasticity after stroke with some promising outcomes reported [6], [7], [9]. Motor representational plasticity is regarded as a neural substrate for rehabilitative training to improve motor recovery [3]. Our finding that iTBS can interact with MT to enhance motor map plasticity points to a possibility that a combination of the brain stimulation and MT can produce synergistic effects to achieve a greater degree of functional recovery for patients with stroke. In the current study, the interaction between MT and iTBS in the modulation of representation plasticity was highly dependent on BDNF Val66Met polymorphism. This suggests that intact BDNF signaling may be required for synergistic outcomes of rehabilitative training and therapeutic brain stimulation and that pharmacological interventions enhancing BDNF function may also positively affect the therapeutic effects of the combinatorial approach.
